# Sodium taurocholate cotransporting polypeptide inhibition efficiently blocks hepatitis B virus spread in mice with a humanized liver

**DOI:** 10.1038/srep27782

**Published:** 2016-06-09

**Authors:** Tasuku Nakabori, Hayato Hikita, Kazuhiro Murai, Yasutoshi Nozaki, Yugo Kai, Yuki Makino, Yoshinobu Saito, Satoshi Tanaka, Hiroshi Wada, Hidetoshi Eguchi, Takeshi Takahashi, Hiroshi Suemizu, Ryotaro Sakamori, Naoki Hiramatsu, Tomohide Tatsumi, Tetsuo Takehara

**Affiliations:** 1 Department of Gastroenterology and Hepatology, Osaka University Graduate School of Medicine, Suita, Osaka, Japan; 2 Department of Gastroenterological Surgery, Osaka University Graduate School of Medicine, Suita, Osaka, Japan; 3 Department of Laboratory Animal Research, Central Institute for Experimental Animals, Kawasaki, Japan

## Abstract

Sodium taurocholate cotransporting polypeptide (NTCP) is a recently discovered hepatitis B virus (HBV) receptor. In the present study, we used TK-NOG mice with a humanized liver to examine the impact of endogenous NTCP expression on HBV infection. Upon inoculation with HBV, these mice exhibited clear viremia in 2 weeks, and serum HBV DNA levels gradually increased. The frequency of HBsAg-positive hepatocytes in the liver was 5.1 ± 0.6% at 2 weeks and increased with increasing HBV DNA levels, reaching 92.9 ± 2.8% at 10 to 12 weeks. *In vivo* siRNA-mediated NTCP knockdown before and after HBV inoculation significantly suppressed the levels of HBV replication and the frequency of HBsAg-positive hepatocytes at 2 weeks, whereas NTCP knockdown 13 weeks after infection did not affect these parameters. Similar to the humanized mouse livers in the early phase of HBV infection, human liver samples from chronic hepatitis B patients, especially those treated with nucleos(t)ide analogues, contained a considerable number of hepatocytes that were negative for the anti-HBs antibody. In conclusion, NTCP inhibition prevents the spread of HBV-infected hepatocytes in mice with a humanized liver. NTCP-targeted therapy has potential for regulating HBV infection in patients with chronic hepatitis B.

Hepatitis B virus (HBV) is one of the most common infectious diseases worldwide. More than 350 million people are infected as chronic carriers and are at risk of developing end-stage liver failure and hepatocellular carcinoma^1^. The goal of HBV treatment is to eliminate the virus by clearing or reducing the levels of covalently closed circular DNA (cccDNA) in infected cells. Current therapies for chronic HBV infection are limited to interferon (IFN) and nucleos(t)ide analogues (NAs). These agents regulate HBV replication but do not achieve the ultimate treatment goal. Therefore, new antiviral therapeutic strategies are required. HBV infects a limited number of species, including humans and chimpanzees^2^, but the use of chimpanzees as an infectious model is ethically restricted. Only specific cells, including primary human hepatocytes (PHHs)^3^ and HepaRG cells^4^, are susceptible to HBV. PHHs are difficult to acquire, and HepaRG cells may lack stable susceptibility depending on the differentiation state. Thus, reliable infection models for analyzing the HBV life cycle do not exist either *in vivo* or *in vitro*, and these limitations hamper HBV research. The use of mice with a humanized liver may resolve these difficulties^5^,^6^.

Several groups have reported that sodium taurocholate cotransporting polypeptide (NTCP) expression renders non-susceptible hepatoma cells permissive to HBV^7^,^8^,^9^. NTCP is therefore expected to be a new therapeutic target in chronic hepatitis B (CHB) infection^10^. However, these studies used hepatoma cells with forced NTCP expression that do not completely exhibit genuine hepatocyte physiology. In addition, HBV is replicated at lower levels in these cells, which reside in a transient infection mode, compared to PHHs^11^. Therefore, NTCP overexpressing hepatoma cells have some limitations to analyze HBV infection.

In the present study, we analyzed the impact of endogenous NTCP expression on HBV infection using TK-NOG mice with a humanized liver, which harbor non-transformed human hepatocytes, and primary hepatocytes isolated from these mice. We found that NTCP inhibition efficiently suppressed the dissemination of HBsAg-positive hepatocytes during the early phase of HBV infection in chimeric mice. On the other hand, NTCP inhibition had no significant impact on serum HBV DNA and HBsAg levels after almost all the hepatocytes were infected with HBV. These findings indicate that NTCP inhibition suppresses the spread of HBV infection in the presence of uninfected hepatocytes. Many HBsAg-negative hepatocytes were detected in the livers of most CHB patients. Together with the finding that the frequency of HBsAg-negative hepatocytes was higher in patients who were taking NAs compared to untreated patients, NTCP inhibition may have therapeutic efficacy in CHB patients, especially those on NAs.

## Results

### PHHs from humanized liver chimeric mice are susceptible to HBV

**Figure 1:**
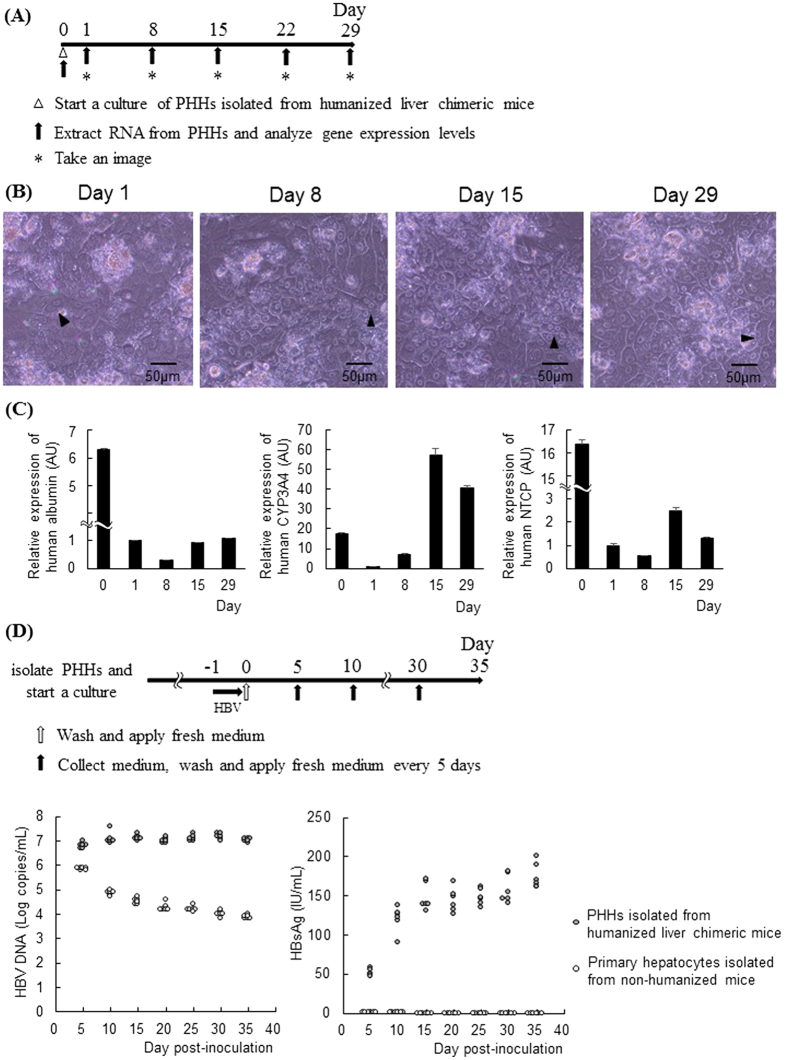
PHHs isolated from humanized liver chimeric mice were susceptible to HBV. (**A–C**) PHHs were isolated from humanized liver chimeric mice. Schematic of experimental procedure (**A**). Representative images of PHHs isolated from humanized liver chimeric mice at the indicated times (**B**). Arrowheads indicate cells with a diploid nucleus. Real-time RT-PCR analysis of gene expression levels at the indicated time (n = 4) (**C**). (**D,E)** PHHs were incubated with HBV inoculum at 500 GEq/cell for 24 hours. Schematic of experimental procedure (**D**). HBV DNA and HBsAg levels in culture media from PHHs isolated from humanized liver chimeric mice or non-humanized mice were quantified every 5 days after HBV inoculation (n = 6) (**E**). AU; arbitrary unit.

PHHs were isolated from humanized -liver chimeric TK-NOG mice and seeded on plates (Fig. 1A). These cells were square and some have a diploid nucleus. The morphology did not significantly change during one month of observation (Fig. 1B). The expression levels of human NTCP, CYP3A4, and albumin decreased one day after seeding, but their expression was maintained for one month (Fig. 1C). After incubation with HBV (500 Genome Equivalent (GEq)/cell) for 24 hours, the PHHs were washed, and fresh culture medium was applied every 5 days (Fig. 1D). HBsAg and HBV DNA levels were detected in the culture medium 5 days after inoculation. HBV DNA levels remained at approximately 7 log copies/ml after HBV inoculation. HBsAg levels gradually increased, reaching 147.8 ± 6.9 IU/ml at 15 days post-inoculation, and remained at approximately 150 IU/ml (Fig. 1E). In contrast, HBV DNA and HBsAg levels decreased in the culture medium of primary hepatocytes isolated from non-humanized liver mice after HBV inoculation (500 GEq/cell) (Fig. 1E). The HBV DNA levels in the culture medium of HBV-inoculated PHHs decreased after treatment with entecavir, whereas those of HBV-inoculated primary murine hepatocytes were unchanged (Supplementary Fig. 1). These data suggested that PHHs isolated from humanized liver chimeric mice were susceptible to HBV and persistently produced HBV for one month.

### NTCP inhibition decreases HBV susceptibility of PHHs

**Figure 2:**
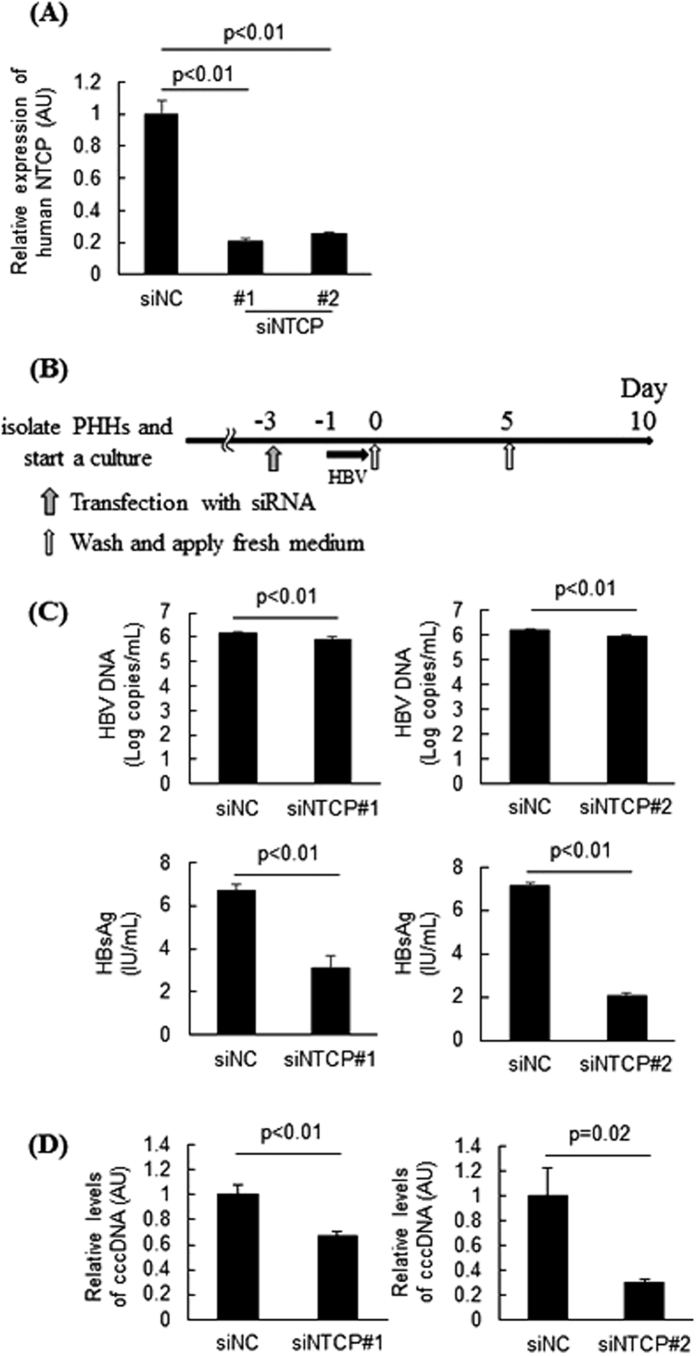
NTCP inhibition decreased susceptibility to HBV. (**A**) PHHs were transfected with specific siRNA against human NTCP. We used two siRNA sequences (siNTCP#1 and siNTCP#2), which target different amino acid sequences within human NTCP. Human NTCP expression levels were determined at 3 days post-transfection (n = 4). (**B–D)** PHHs were inoculated with HBV 3 days after transfection with siRNA against NTCP. Schematic of experimental procedure (**B**). HBV DNA and HBsAg levels in the culture medium at 10 days post-inoculation (n = 4) (**C**). cccDNA levels in PHHs at 10 days post-inoculation (n = 4) (**D**). AU; arbitrary unit.

PHHs were transfected with siRNA against human-specific NTCP to examine the effect of NTCP inhibition on HBV infection *in vitro*. The siRNA-mediated knockdown of NTCP successfully suppressed its expression level (Fig. 2A). PHHs were transfected with siRNA and then incubated with HBV (50 GEq/cell) for 24 hours. The PHHs were washed, and fresh culture medium was applied every 5 days after inoculation (Fig. 2B). At 10 days after inoculation, HBsAg and HBV DNA levels in the culture medium (Fig. 2C) and cccDNA levels in the PHHs (Fig. 2D) were significantly lower in the NTCP knockdown group than in the control siRNA-treated group. These data suggested that NTCP inhibition decreased the susceptibility of PHHs to HBV.

### HBV-infected hepatocytes disseminated after HBV inoculation in chimeric mice

**Figure 3:**
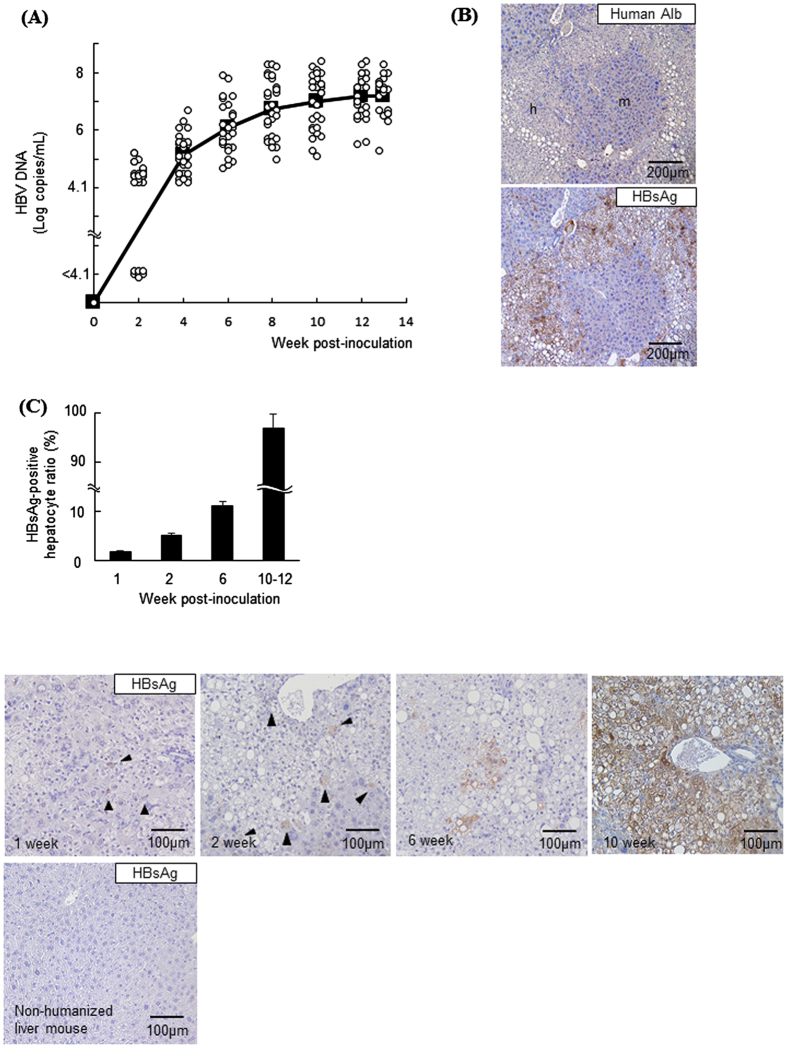
HBV-infected hepatocytes disseminated in chimeric mouse livers. Humanized liver chimeric mice were inoculated with HBV (1.0 × 10^6.1^ copies). (**A**) Serum HBV DNA levels were measured after inoculation (n = 29). Open circles represent individual HBV DNA levels at the indicated point, closed squares represent the average HBV DNA level at the indicated point. (**B**) Representative images of human albumin and HBsAg immunohistochemical analysis of serial liver sections at 10 weeks post-inoculation. ‘m’ = mouse; ‘h’ = human. (**C**) Representative images of HBsAg immunohistochemical analysis and the ratio of HBsAg-positive cells in the livers at the indicated week post-inoculation (n = 3). Arrowheads indicate HBsAg-positive hepatocytes.

Humanized liver chimeric TK-NOG mice were intravenously inoculated with HBV (1.0 × 10^6.1^ copies). Serum HBV DNA was detected beginning at 2 weeks post-inoculation, and the levels gradually increased, reaching approximately 7 log copies/ml at 8 weeks post-inoculation. Afterward, the levels remained at approximately 7 log copies/ml (Fig. 3A). At 10 to 12 weeks post-inoculation, almost all human albumin-positive hepatocytes were positive for HBsAg (Fig. 3B). A time-course analysis revealed that the frequency of HBsAg-positive hepatocytes was 1.7 ± 0.3% at 1 week post-inoculation and 5.1 ± 0.6% at 2 weeks post-inoculation. This frequency gradually increased with increasing serum HBV DNA levels, reaching 92.9 ± 2.8% when serum HBV DNA levels plateaued (Fig. 3C). Overall, our *in vivo* model showed the spread of HBV infection over time after HBV inoculation.

### NTCP inhibition blocks the spread of HBV-infected hepatocytes *in vivo*

**Figure 4:**
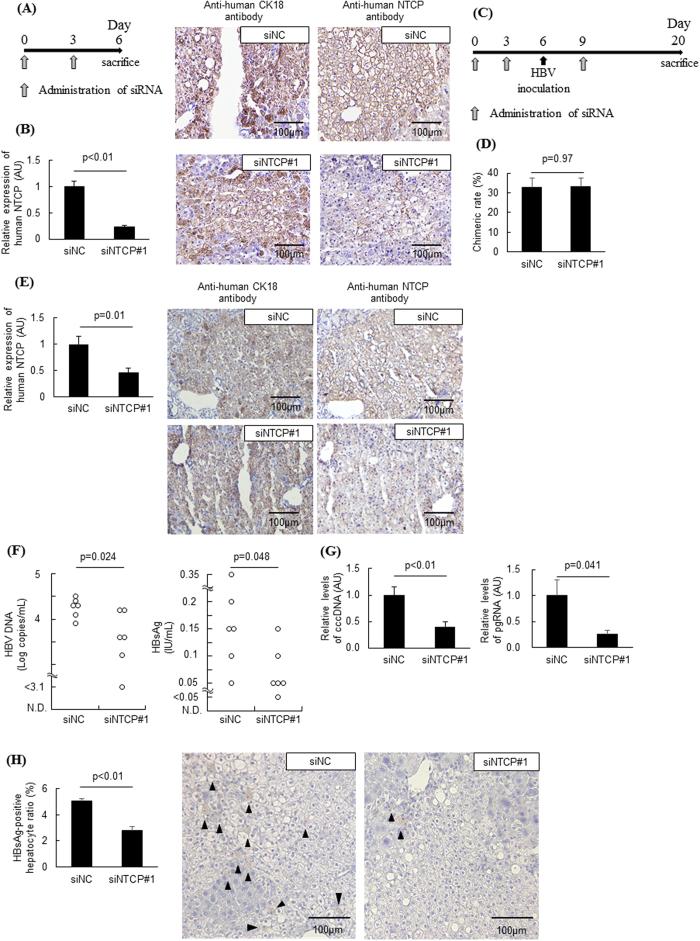
NTCP inhibition suppressed the spread of HBV infection *in vivo*. (**A,B)** Humanized liver chimeric mice were transfected twice with siRNA against human-specific NTCP. Liver specimens were immunostained with anti-human-specific CK18 and NTCP antibodies. CK18 is expressed in hepatocytes, and positive staining for human CK18 indicated the presence of human hepatocytes. Schematic of experimental procedure (**A**). Human NTCP expression levels (n = 5, each spot is from 1 chimeric mouse) and representative images of NTCP and CK-18 immunohistochemical staining of serial liver sections (**B**). (**C–H**) Humanized liver chimeric mice were transfected with siRNA against human-specific NTCP before and after HBV inoculation and sacrificed 2 weeks after inoculation (n = 6). Schematic of experimental procedure (**C**). The chimeric rates in each group at the time of HBV inoculation (**D**). NTCP expression levels (**E**). Serum HBV DNA and HBsAg levels were compared using the Mann-Whitney U test (**F**). cccDNA and pgRNA levels in hepatocytes (**G**). The ratio of HBsAg-positive human hepatocytes and representative images of HBsAg immunohistochemical staining. Arrowheads indicate HBsAg-positive hepatocytes (**H**). AU; arbitrary unit.

To examine the impact of NTCP inhibition on HBV infection *in vivo*, human NTCP expression was knocked down *in vivo* using siRNA against human-specific NTCP. We confirmed that this siRNA efficiently decreased human NTCP mRNA levels in the chimeric mouse liver and suppressed NTCP expression in human hepatocytes (Fig. 4A,B). Humanized liver chimeric mice were randomly assigned to the NTCP knockdown group or the negative control group and were injected with the appropriate siRNA before and after HBV inoculation. Mice were sacrificed 20 days after the first siRNA administration (Fig. 4C). No significant difference was observed in the chimeric rates between the NTCP knockdown group and the negative control group at HBV inoculation (Fig. 4D). NTCP mRNA levels in the chimeric mouse liver and NTCP expression in human hepatocytes remained suppressed in the NTCP knockdown group at the time of sacrifice with no significant changes in the serum levels of total bile acids and liver functions (Fig. 4E, Supplementary Fig. 2). Serum HBV DNA and HBsAg levels were significantly lower in the NTCP knockdown group than in the negative control group (Fig. 4F). Significant reductions were also observed in cccDNA and pregenome RNA (pgRNA) levels in the liver of NTCP knockdown mice (Fig. 4G). The frequency of HBsAg-positive human hepatocytes was significantly lower in the NTCP knockdown group than in the control group (Fig. 4H). These results suggested that NTCP inhibition suppressed the spread of HBV-infected hepatocytes in humanized liver chimeric mice and evoked declines in serum HBV DNA and HBsAg levels.

### The anti-HBV effect of NTCP inhibition is not observed in late-stage HBV infection in chimeric mice

**Figure 5:**
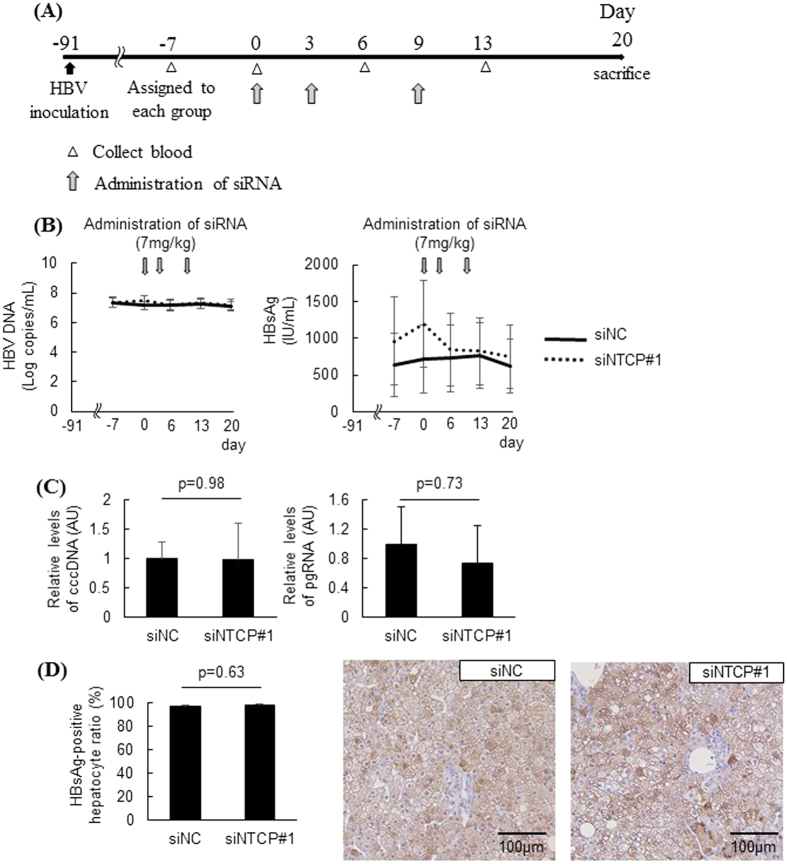
NTCP inhibition in HBV-infected chimeric mice did not affect HBV infection. HBV-infected humanized -liver chimeric mice were treated with siRNA against human-specific NTCP 13 weeks after inoculation (n = 5). (**A**) Schematic of experimental procedure. (**B**) Serum HBV DNA and HBsAg levels. (**C**) Liver cccDNA and pgRNA levels 20 days after the first siRNA administration. (**D**) Representative images of HBsAg immunohistochemical analysis 20 days after the first siRNA administration and the ratio of HBsAg-positive cells (n = 5). AU; arbitrary unit.

Humanized liver chimeric mice that had been inoculated with HBV 13 weeks earlier were challenged with NTCP inhibition to determine its effect on HBV-infected hepatocytes (Fig. 5A). Chimeric mice were randomly assigned to the NTCP knockdown group or the negative control group, and each siRNA was administered to the mice three times (Fig. 5A). No significant differences were observed in serum HBV DNA, serum HBsAg, liver cccDNA, or liver pgRNA levels between the NTCP knockdown group and the negative control group (Fig. 5B,C). The two groups showed no significant difference in the frequency of HBsAg-positive hepatocytes, which was approximately 100% (Fig. 5D).

### HBsAg-negative hepatocytes are substantially observed in human liver samples from CHB patients

**Figure 6:**
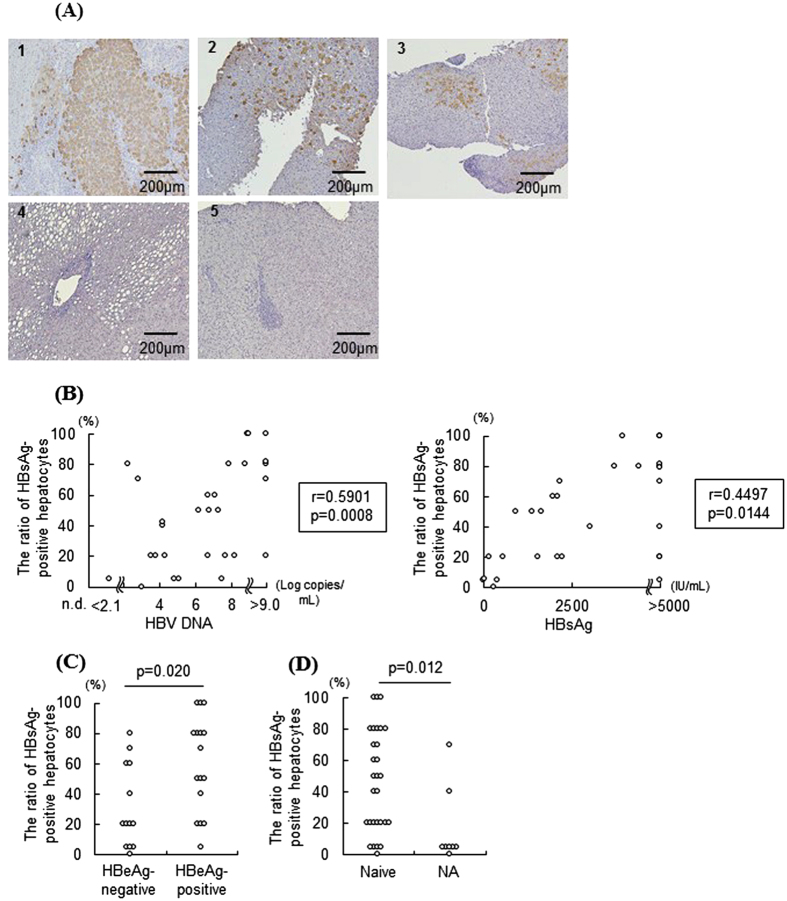
Liver samples from untreated CHB patients contained HBsAg-negative hepatocytes. Liver specimens obtained from 37 CHB patients (29 untreated CHB patients and 8 CHB patients on NAs) were stained with an anti-HBs antibody. (**A**) Representative images of liver specimens from patients with untreated CHB, non-alcoholic steatohepatitis or chronic hepatitis C (Table 2). (**B**) Pearson’s correlation coefficient was used to analyze the relation of the frequency of HBsAg-positive hepatocytes with serum HBV DNA or HBsAg levels in untreated CHB patients. HBV DNA levels that were detectable but below the lower limit of detection, which is 2.1, were given a value of 2.0. HBV DNA levels above the upper limit of limitation, which is 9.0, were given a value of 9.1. HBsAg levels above the upper limit of detection, which is 5000, were valued at 5001. (**C**) Comparison of the frequency of HBsAg-positive hepatocytes between HBeAg-negative (n = 13) and HBeAg-positive untreated CHB patients (n = 16) (Mann-Whitney U test). (**D**) Comparison of the frequency of HBsAg-positive hepatocytes in untreated CHB patients (naïve, n = 29) and in CHB patients on NAs (NA, n = 8) (Mann-Whitney U test).

Our findings with the chimeric mice suggested that NTCP inhibition conferred anti-HBV effects in the presence of uninfected hepatocytes in the liver. To evaluate the potential clinical efficacy of NTCP inhibition, we analyzed the frequency of HBV-infected hepatocytes in 37 CHB patients, including 29 treatment-naïve patients and 8 patients being treated with NAs (Table 1). Almost all the untreated CHB patients harbored HBsAg-negative hepatocytes in their livers, and the distribution of HBsAg-positive cells was scattered or clustered (Fig. 6A), which was consistent with previous reports^12^,^13^,^14^. The frequency of HBsAg-positive hepatocytes was positively correlated with serum HBV DNA and HBsAg levels in treatment-naïve CHB patients (Fig. 6B) and significantly higher in HBeAg-positive patients than in HBeAg-negative patients (Fig. 6C). CHB patients who were taking NAs had a significantly lower frequency of HBsAg-positive hepatocytes (predominantly 5% or less) compared to untreated CHB patients (Fig. 6D).

## Discussion

In the present study, we used chimeric TK-NOG mice with a humanized liver as an *in vivo* model of HBV infection. Serum HBV DNA levels gradually increased in chimeric mice after HBV inoculation. Chimeric mice, which are susceptible to HBV, have recently begun to be used to study HBV pathobiology. However, the mode of HBV infection has not been extensively studied. In the present study, we clarified that a small number of HBV-infected cells was detected at 1 week post-inoculation by immunohistochemistry. The frequency of HBsAg-positive hepatocytes increased with increasing serum HBV DNA levels. At 10 to 12 weeks post-inoculation, serum HBV DNA levels plateaued and the HBsAg-positive hepatocyte frequency was greater than 90% (Fig. 3B,C). The rate of HBsAg-positive hepatocytes in chimeric mice at 10 to 12 weeks post-inoculation was quite different from that in livers from CHB patients, who contained many HBsAg-negative hepatocytes (Fig. 6A). Previous reports have noted that HBV-infected hepatocytes are eliminated by host immune responses, followed by the regeneration of naïve hepatocytes in humans^15^. Because TK-NOG mice lack B cells, T cells, and natural killer cells, HBV-infected human hepatocytes are not able to be eliminated, causing that HBsAg-negative hepatocytes hardly exist at the later time point. In this aspect, HBV-infected humanized liver chimeric TK-NOG mice whose serum HBV DNA levels plateau do not recapitulate a mode of infection in CHB patients. This discrepancy could be solved by the development of immunocompetent humanized liver chimeric mice^16^. Nonetheless, during the early phase of infection of this model, it is possible to observe a situation where the infection of HBV is to expand. In this phase, HBV-infected humanized -liver chimeric TK-NOG mice can be used to examine the current HBV infection to uninfected hepatocytes as seen in the clinical settings.

The entry of HBV into hepatocytes is a candidate therapeutic target in HBV infectious diseases. With respect to HBV factors, the viral component of the preS1 domain in the large envelope protein (L-protein) was reported to be essential for entry into hepatocytes^17^. Myrcludex B, a synthetic lipopeptide derived from the preS1 domain of the HBV L-protein, blocked the entry of hepatitis B virus both *in vitro*^18^,^19^ and *in vivo*^20^,^21^. With respect to host factors, NTCP was recently identified as a candidate receptor for HBV entry^22^. And later, Myrcludex B was confirmed to bind NTCP and inhibit the function of NTCP^7^,^23^. However, it is unclear whether the effect of Myrcludex B on HBV infection depends on NTCP alone. Some studies using HepaRG cells or PHHs previously reported that NTCP inhibition reduced the susceptibility to HBV^10^,^24^, but the *in vivo* effect of this inhibition still remained unclear. The present study constitutes the first direct demonstration that NTCP inhibition can suppress the spread of HBV-infected hepatocytes *in vivo*.

In the present study, we demonstrated that siRNA-mediated NTCP knockdown suppressed the spread of HBV-infected hepatocytes in the presence of uninfected hepatocytes. In contrast, we determined that NTCP inhibition did not affect HBV replication or persistent infection in chimeric mice when almost all hepatocytes were infected with HBV (Fig. 5B–D). However, as described in the first paragraph of the Discussion section, the frequency of HBsAg-positive hepatocytes was quite different between HBV-infected chimeric mice in the HBV DNA plateau phase and CHB patients. In patients with CHB, hepatocytes follow a particular cycle: HBV-infected hepatocytes are eliminated by host immune responses, naïve hepatocytes are regenerated, and HBV then infects the regenerated hepatocytes^15^. The ratio of HBsAg-positive hepatocytes in CHB patients is probably regulated by each speed of this cycle. In fact, HBsAg-negative hepatocytes were consistently observed in liver samples from most CHB patients (Fig. 6A–C). In particular, all CHB patients taking NAs harbored many HBsAg-negative hepatocytes, and their HBsAg-positive hepatocyte ratio was lower than that in untreated CHB patients (Fig. 6D). Collectively, our *in vivo* results suggested the potential for treating CHB patients with NTCP-targeted therapy, which could possibly inhibit the infection of naïve hepatocytes. Conceivably, NTCP-targeted therapy may not be effective in the few patients whose HBsAg-positive hepatocyte ratio is near 100%. For these patients, combination therapy with NAs might be a good choice. Further studies are necessary to confirm these hypotheses.

It is generally reported that there are 2 pathways that supply additional cccDNA into the nucleus in HBV-infected cells^25^. One, HBV released from hepatocytes can re-infect the same hepatocytes. Two, HBV nucleocapsid in cytoplasmic region may redirect to nucleus. Both pathways could provide a template of HBV replication into the nucleus. The former requires entry receptors, but the latter does not. The present study revealed that NTCP inhibition did not affect HBV antigenemia, viremia, or the hepatic levels of an RNA intermediate (pgRNA) and an intracellular viral replication template (cccDNA) in HBV-infected chimeric mice at 13 weeks post-inoculation, when almost all hepatocytes were infected with HBV (Fig. 5). Although it is unclear whether additional cccDNA supplement is necessary to persistent virus infection, our results suggested that at least, re-infection has less impact on persistent HBV infection.

[Table t2]Some researchers have shown that PHHs isolated from humanized liver chimeric uPA/SCID mice were able to be cultured *in vitro* over a month and susceptible to HBV^11^,^26^. The present study revealed that PHHs isolated from humanized liver chimeric TK-NOG mice were similarly maintained over a month and susceptible to HBV leading to persistent infection. PHHs isolated from humanized liver chimeric TK-NOG mice required only 50 or 500 GEq/cell of HBV for *in vitro* research of HBV-infected disease, while HepaRG cells^27^ or NTCP-overexpressing hepatoma cells^9^ required 1.25 × 10^4^ or 6.0 × 10^3^ GEq/cell of HBV. Our results from siRNA-mediated knockdown experiments using PHHs well reflected those using humanized liver TK-NOG mice. Taken together, PHHs isolated from humanized liver chimeric TK-NOG mice are useful for studying HBV.

Additionally, we successfully performed siRNA-mediated knockdown in human hepatocytes in humanized liver chimeric mice using Invivofectamine (Thermo Fisher Scientific). To the best of our knowledge, this is the first study to report that human hepatocytes in humanized -liver chimeric mice can be transfected with siRNA. We suppressed NTCP mRNA expression levels by more than half for at least 2 weeks using siRNA transfection (Fig. 4B,E). Because this technique can be used to knockdown the expression of a target gene of human hepatocytes in chimeric mice in a rapid and straightforward manner, it will be useful for further examinations of specific genes involved in HBV infection and of other human-specific infectious viruses, such as hepatitis C virus.

In conclusion, NTCP inhibition suppressed the *in vivo* spread of HBV infection in the presence of uninfected hepatocytes. Livers in CHB patients contained varying degrees of HBsAg-negative hepatocytes, as evidenced by immunohistochemistry. Taken together, our data suggest that NTCP is a potential new target for the treatment of CHB.

## Materials and Methods

### Generation of a humanized liver TK-NOG mouse model

A humanized liver chimeric TK-NOG mouse model (NOD/Shi-scid IL-2 Rγ^null^ mice expressing a herpes simplex virus type 1 thymidine kinase transgene under regulation of the albumin gene promoter) was generated as previously described^28^. The human hepatocyte chimeric rate correlated with serum human albumin levels^28^, which were measured using the Human Albumin ELISA Quantitation Set (E80-129, Bethyl Laboratories Inc., Montgomery, TX). The serum levels of alanine transaminase, alkaline phosphatase, conjugated bilirubin and total bile acids were measured at the Nagahama Laboratory of Oriental Yeast Co., Ltd. (Tokyo, Japan). Humanized liver chimeric mice were maintained in a specific pathogen-free facility and treated humanely. All mouse studies were conducted in strict accordance with the Guide for the Care and Use of Laboratory Animals from Osaka University Medical School and the Central Institute for Experimental Animals (CIEA). All experimental protocols were approved by the Animal Care and Use Committee of Osaka University Medical School and the Animal Care Committee of the CIEA.

### Isolation and culture of PHHs from humanized liver chimeric mice

PHHs were isolated from chimeric mice with estimated chimeric liver rates of greater than 35% using two-step collagenase-pronase liver perfusion as previously described^29^. PHHs were seeded on type I collagen-coated plates (AGC Techno Glass CO., LTD., Shizuoka, Japan). Culture medium containing 2% DMSO (Sigma-Aldrich, St. Louis, MO) was changed every five days. All experiments were initiated within 10 days after isolating cells from chimeric mice. In some experiments, PHHs were treated with 1000 ng/ml entecavir (Sigma-Aldrich).

### HBV inoculum preparation and HBV infection

The culture supernatant of HepG2.2.15, which produces HBV Genotype D^30^, was used as the HBV inoculum for the *in vitro* experiments. Sera from a patient with CHB (Genotype C, 9.1 log copies/ml) was used for the *in vivo* experiments under approval from the Institutional Review Board for Clinical Research at Osaka University Hospital (12050). Written informed consent was obtained from this patient. The methods were carried out in accordance with the approved guidelines. HepG2.2.15 culture supernatant was collected every 3 days, passed through a 0.45-μm filter (Merck Millipore, Billerica, MA), and concentrated approximately 200-fold using PEG-it Virus Precipitation Solution (System Biosciences, Mountain View, CA). PHHs were treated with HBV inoculum for 24 hours in the presence of 4% polyethylene glycol 8000 (Promega, Madison, WI). After incubation with HBV inoculum, the cells were washed three times with PBS containing 2% DMSO. The patient sera was diluted to 7.1 log copies/ml. Humanized -liver chimeric mice with an estimated chimeric rate of greater than 20% were injected intravenously with 100 μl of the diluted sera. Blood was collected from an external jugular vein.

### Transfection of siRNA against NTCP

Two siRNAs against human NTCP, NTCP_#1 (s224646) and NTCP_#2 (s13033), and the appropriate negative controls (*in vitro*: #4390843; *in vivo*: #4457289) were purchased from Ambion. *In vitro* transfections were performed using Lipofectamine RNAiMAX (Thermo Fisher Scientific, Waltham, MA) according to the manufacturer’s protocol. Invivofectamine 2.0 Reagent (Thermo Fisher Scientific) was used for *in vivo* transfections according to the manufacturer’s protocol. Briefly, Invivofectamine 2.0 Reagent-siRNA complexes (7 mg/kg) dialyzed using a Float-A-Lyzer G2 (Spectrum Laboratories, Rancho Dominguez, CA) were injected into chimeric mice (10 μl/g) via the tail vein.

### Measurement of HBsAg and HBV DNA levels

HBsAg levels were measured in the *in vitro* samples and in each fold dilution of the *in vivo* serum samples using a chemiluminescent immunoassay (CLIA System, Abbott Laboratories, North Chicago, IL; lower limit of detection = 0.01 IU/ml). HBV DNA levels were measured using the COBAS TaqMan HBV Test (Roche Diagnostics, Switzerland) with a lower limit of detection of 2.1 log copies/ml. The samples were diluted 10-fold (*in vitro* experiments) or 10- or 100-fold (*in vivo* experiments) for analysis.

### RNA extraction and real-time reverse transcription PCR (RT-PCR)

Total RNA was prepared using the RNeasy Mini Kit (Qiagen, Valencia, CA) according to the manufacturer’s protocol. Prepared RNA was treated with DNase using the TURBO DNA-free Kit (Ambion, Austin, TX) to analyze pgRNA. For cDNA synthesis, template RNA was reverse transcribed using ReverTra Ace qPCR RT Master Mix (TOYOBO, Tokyo, Japan). Real-time RT-PCR was performed using the QuantStudio 6 Flex Standard Real-Time System (Applied Biosystems, Foster City, CA). The following TaqMan Gene Expression Assays were used: human β-actin (Hs99999903_m1, Ambion) and human NTCP (Hs00914888_m1, Ambion). To detect pgRNA, the primer set and probe were designed as follows: primer set, 5′-TGTCCTACTGTTCAAGCCTCCAA-3′ (1855-1877) and 5′-GAGAGTAACTCCACAGTAGCTCCAA-3′ (1928–1952); probe, 5′-FAM-CATGGACATCGACCC-3′ (1902–1916). All target gene expression levels were normalized to the quantified expression levels of human β-actin mRNA.

### DNA extraction and real-time PCR for HBV cccDNA

Total DNA was prepared using the QIAamp DNA Mini Kit (Qiagen) according to the manufacturer’s protocol. To detect Genotype D cccDNA, the primer set and probe were designed according to a previous study^31^: primer set, 5′-CGTCTGTGCCTTCTCATCTGC-3′ (1552–1572) and 5′-GCACAGCTTGGAGGCTTGAA-3′ (1865–1884); probe, 5′-FAM-CTGTAGGCATAAATTGGT-3′ (1783–1800). PCR was performed at 50 °C for 2 minutes, 95 °C for 10 minutes, and 50 cycles of 95 °C for 15 seconds and 60 °C for 1 minute. To detect Genotype C cccDNA, the following primer set and probe were used: primer set, 5′-TCCCCGTCTGTGCCTTCTC-3′ (1420–1438) and 5′-GCACAGCTTGGAGGCTTGA-3′ (1738–1756); probe, 5′-FAM-CCGTGTGCACTTCG-3′ (1449–1462). PCR was performed at 50 °C for 2 minutes, 94 °C for 10 minutes, and 50 cycles of 94 °C for 30 seconds and 60 °C for 90 seconds. Real-time PCR was performed using the QuantStudio 6 Flex System (Applied Biosystems). Target gene expression levels were normalized to the quantified expression levels of human RNase P using the TaqMan Copy Number Reference Assay (#4403326, Thermo Fisher Scientific).

### Immunostaining

Cryosections (6 μm) from humanized -liver chimeric mice were immunostained using an anti-human NTCP antibody (HPA042727, Sigma-Aldrich) and an anti-human cytokeratin (CK) 18 antibody (NB110-56910, Novus Biologicals, Littleton, CO). Formalin-fixed and paraffin-embedded liver sections (3 μm) were immunostained using an anti-HBs antibody (bs-1557 G, Bioss, Woburn, MA) and an anti-human albumin antibody (A80-129 A, Bethyl Laboratories Inc.).

### Human liver samples

Resected liver samples were obtained from hepatocellular carcinoma patients infected with or without HBV at the time of operation, as approved by the Institutional Review Board for Clinical Research at Osaka University Hospital (15267). Written informed consent was obtained from each patient. The methods were carried out in accordance with the approved guidelines. Non-tumor-containing sections of surgically resected specimens were used. Liver biopsy samples were obtained from patients infected with or without HBV, as approved by the Institutional Review Board for Clinical Research at Osaka University Hospital (15267), and written informed consent was obtained from each patient. The methods were carried out in accordance with the approved guidelines. Residual liver biopsy specimens were used. Liver samples were stained with an anti-HBs antibody and classified according to the frequency of HBsAg-positive hepatocytes into the following categories: 0, 5, 10, 20, 30, 40, 50, 60, 70, 80, 90, or 100%.

### Statistical analysis

The data are presented as scatter charts or as the mean ± standard error. When the data exhibited a normal distribution, the differences between two groups were determined using Student’s t test. Otherwise, the differences between two groups were compared using appropriate statistical methods, which are documented in the figure legends. A value of P < 0.05 was considered to indicate statistical significance.

All the *in vitro* experiments were repeated three times using PHHs isolated from three different humanized -liver chimeric mice, and similar results were obtained in each experiment. Representative data from a single experiment are presented.

## Additional Information

**How to cite this article**: Nakabori, T. *et al*. Sodium taurocholate cotransporting polypeptide inhibition efficiently blocks hepatitis B virus spread in mice with a humanized liver. *Sci. Rep*. **6**, 27782; doi: 10.1038/srep27782 (2016).

## Supplementary Material

Supplementary Information

## Figures and Tables

**Table 1:** Baseline patient characteristics.

Characteristic		Naïve	NA	p
N		29	8	
Age (years)	Median	44	56.5	p = 0.0860^a^
	25^th^–75^th^ percentile	35–61	48.25–66.5	
Sex				
Male/Female	N	15/14	6/2	p = 0.2394^b^
Material				
Resected/Biopsy	N	5/24	3/5	p = 0.2178^b^
HBV DNA (log copies/ml)	Range	<2.1 to >9.0	n.d. −3.2	p < 0.0001^a^
HBsAg (IU/l)	Range	0.61 to >10000	36.84–3146	p = 0.0966^a^
HBeAg				
Positive/Negative	N	16/13	3/5	p = 0.7949^b^
ALT (U/l)	Median	42	18.5	p = 0.0018^a^
	25^th^–75^th^ percentile	31.5–107	13.25–32.75	
Medication history				
LAM + ADV/ETV	N		2/6	
Period (months)	Median		39	
	25^th^–75^th^ percentile		10.5–93.5	

LAM: lamivudine n.d.: not detected. ADV: adefovir dipivoxil. ETV: entecavir.

^a^Mann-Whitney U test.

^b^Pearson chi-squared test.

**Table 2 t2:** Clinical background of the patients.

Pt	Sample	Age (years)	Sex	HBV DNA (log copies/ml)	HBsAg (IU/l)	HBeAg	ALT (U/l)	NAs	Percentage of HBsAg-positive hepatocytes (%)
1	resected	49	m	7.9	3714	positive	77	-	80
2	biopsy	67	f	4.2	3033	negative	19	-	40
3	biopsy	34	m	3.8	1541	negative	32	-	20
4 (NASH)	resected	76	m	–	–	–	68	–	–
5 (chronic hepatitis C)	biopsy	69	f	–	–	–	19	–	–

NASH: Non-alcoholic steatohepatitis.

## References

[b1] Trépo C., Chan H. L. & Lok A. Hepatitis B virus infection. Lancet 384, 2053–2063, doi: 10.1016/S0140-6736(14)60220-8 (2014). 24954675

[b2] Kim S. H., Oh H. K., Ryu C. J., Park S. Y. & Hong H. J. *In vivo* hepatitis B virus-neutralizing activity of an anti-HBsAg humanized antibody in chimpanzees. Exp Mol Med 40, 145–149, doi: 10.3858/emm.2008.40.1.145 (2008). 18305407 PMC2679323

[b3] Galle P. R. . *In vitro* experimental infection of primary human hepatocytes with hepatitis B virus. Gastroenterology 106, 664–673 (1994). 8119538 10.1016/0016-5085(94)90700-5

[b4] Hantz O. . Persistence of the hepatitis B virus covalently closed circular DNA in HepaRG human hepatocyte-like cells. J Gen Virol 90, 127–135, doi: 10.1099/vir.0.004861-0 (2009). 19088281

[b5] Bissig K. D. . Human liver chimeric mice provide a model for hepatitis B and C virus infection and treatment. J Clin Invest 120, 924–930, doi: 10.1172/JCI40094 (2010). 20179355 PMC2827952

[b6] Vanwolleghem T. . Factors determining successful engraftment of hepatocytes and susceptibility to hepatitis B and C virus infection in uPA-SCID mice. J Hepatol 53, 468–476, doi: 10.1016/j.jhep.2010.03.024 (2010). 20591528

[b7] Ni Y. . Hepatitis B and D viruses exploit sodium taurocholate co-transporting polypeptide for species-specific entry into hepatocytes. Gastroenterology 146, 1070–1083, doi: 10.1053/j.gastro.2013.12.024 (2014). 24361467

[b8] Nkongolo S. . Cyclosporin A inhibits hepatitis B and hepatitis D virus entry by cyclophilin-independent interference with the NTCP receptor. J Hepatol 60, 723–731, doi: 10.1016/j.jhep.2013.11.022 (2014). 24295872

[b9] Iwamoto M. . Evaluation and identification of hepatitis B virus entry inhibitors using HepG2 cells overexpressing a membrane transporter NTCP. Biochem Biophys Res Commun 443, 808–813, doi: 10.1016/j.bbrc.2013.12.052 (2014). 24342612

[b10] Watashi K. . Cyclosporin A and its analogs inhibit hepatitis B virus entry into cultured hepatocytes through targeting a membrane transporter, sodium taurocholate cotransporting polypeptide (NTCP). Hepatology 59, 1726–1737, doi: 10.1002/hep.26982 (2014). 24375637 PMC4265264

[b11] Ishida Y. . Novel robust *in vitro* hepatitis B virus infection model using fresh human hepatocytes isolated from humanized mice. Am J Pathol 185, 1275–1285, doi: 10.1016/j.ajpath.2015.01.028 (2015). 25791527

[b12] Wang H. C. . Different types of ground glass hepatocytes in chronic hepatitis B virus infection contain specific pre-S mutants that may induce endoplasmic reticulum stress. Am J Pathol 163, 2441–2449, doi: 10.1016/S0002-9440(10)63599-7 (2003). 14633616 PMC1892360

[b13] Tsai H. W. . A clustered ground-glass hepatocyte pattern represents a new prognostic marker for the recurrence of hepatocellular carcinoma after surgery. Cancer 117, 2951–2960, doi: 10.1002/cncr.25837 (2011). 21692054

[b14] Cheng P. N. . HBsAg expression of liver correlates with histological activities and viral replication in chronic hepatitis B. Ann Hepatol 13, 771–780 (2014). 25332263

[b15] Dandri M. & Petersen J. Hepatitis B virus cccDNA clearance: killing for curing? Hepatology 42, 1453–1455, doi: 10.1002/hep.20976 (2005). 16317676

[b16] Shultz L. D., Brehm M. A., Garcia-Martinez J. V. & Greiner D. L. Humanized mice for immune system investigation: progress, promise and challenges. Nat Rev Immunol 12, 786–798, doi: 10.1038/nri3311 (2012). 23059428 PMC3749872

[b17] Schulze A., Schieck A., Ni Y., Mier W. & Urban S. Fine mapping of pre-S sequence requirements for hepatitis B virus large envelope protein-mediated receptor interaction. J Virol 84, 1989–2000, doi: 10.1128/JVI.01902-09 (2010). 20007265 PMC2812397

[b18] Gripon P., Cannie I. & Urban S. Efficient inhibition of hepatitis B virus infection by acylated peptides derived from the large viral surface protein. J Virol 79, 1613–1622, doi: 10.1128/JVI.79.3.1613-1622.2005 (2005). 15650187 PMC544121

[b19] Glebe D. . Mapping of the hepatitis B virus attachment site by use of infection-inhibiting preS1 lipopeptides and tupaia hepatocytes. Gastroenterology 129, 234–245 (2005). 16012950 10.1053/j.gastro.2005.03.090

[b20] Petersen J. . Prevention of hepatitis B virus infection *in vivo* by entry inhibitors derived from the large envelope protein. Nat Biotechnol 26, 335–341, doi: 10.1038/nbt1389 (2008). 18297057

[b21] Volz T. . The entry inhibitor Myrcludex-B efficiently blocks intrahepatic virus spreading in humanized mice previously infected with hepatitis B virus. J Hepatol 58, 861–867, doi: 10.1016/j.jhep.2012.12.008 (2013). 23246506

[b22] Yan H. . Sodium taurocholate cotransporting polypeptide is a functional receptor for human hepatitis B and D virus. Elife 1, e00049, doi: 10.7554/eLife.00049 (2012). 23150796 PMC3485615

[b23] Slijepcevic D. . Impaired uptake of conjugated bile acids and hepatitis b virus pres1-binding in na(+) -taurocholate cotransporting polypeptide knockout mice. Hepatology 62, 207–219, doi: 10.1002/hep.27694 (2015). 25641256 PMC4657468

[b24] Tsukuda S. . Dysregulation of retinoic acid receptor diminishes hepatocyte permissiveness to hepatitis B virus infection through modulation of sodium taurocholate cotransporting polypeptide (NTCP) expression. J Biol Chem 290, 5673–5684, doi: 10.1074/jbc.M114.602540 (2015). 25550158 PMC4342479

[b25] Grimm D., Thimme R. & Blum H. E. HBV life cycle and novel drug targets. Hepatol Int 5, 644–653, doi: 10.1007/s12072-011-9261-3 (2011). 21484123 PMC3090558

[b26] Hayakawa M. . Development of novel hepatitis B virus capsid inhibitor using in silico screening. Biochem Biophys Res Commun 463, 1165–1175, doi: 10.1016/j.bbrc.2015.06.077 (2015). 26095852

[b27] Schulze A., Mills K., Weiss T. S. & Urban S. Hepatocyte polarization is essential for the productive entry of the hepatitis B virus. Hepatology 55, 373–383, doi: 10.1002/hep.24707 (2012). 21953491

[b28] Hasegawa M. . The reconstituted ‘humanized liver’ in TK-NOG mice is mature and functional. Biochem Biophys Res Commun 405, 405–410, doi: 10.1016/j.bbrc.2011.01.042 (2011). 21238430 PMC3648850

[b29] Hikita H. . Activation of the Mitochondrial Apoptotic Pathway Produces Reactive Oxygen Species and Oxidative Damage in Hepatocytes That Contribute to Liver Tumorigenesis. Cancer Prev Res (Phila) 8, 693–701, doi: 10.1158/1940-6207.CAPR-15-0022-T (2015). 26038117

[b30] Sells M. A., Chen M. L. & Acs G. Production of hepatitis B virus particles in Hep G2 cells transfected with cloned hepatitis B virus DNA. Proc Natl Acad Sci USA 84, 1005–1009 (1987). 3029758 10.1073/pnas.84.4.1005 PMC304350

[b31] Mason A. L., Xu L., Guo L., Kuhns M. & Perrillo R. P. Molecular basis for persistent hepatitis B virus infection in the liver after clearance of serum hepatitis B surface antigen. Hepatology 27, 1736–1742, doi: 10.1002/hep.510270638 (1998). 9620351

