# Supplementary material for: Sodium taurocholate cotransporting polypeptide inhibition efficiently blocks hepatitis B virus spread in mice with a humanized liver
Source: Sci Rep. 2016 Jun 9;6:27782. doi: 10.1038/srep27782 (PMC4899802; doi:10.1038/srep27782)
Supplement: Supplementary Information [file srep27782-s1.pdf]

Supplementary Information

Sodium taurocholate cotransporting polypeptide inhibition efficiently blocks hepatitis B virus spread in mice with a humanized liver.

Tasuku Nakabori[1], Hayato Hikita[1], Kazuhiro Murai[1], Yasutoshi Nozaki[1], Yugo Kai[1], Yuki Makino[1], Yoshinobu Saito[1], Satoshi Tanaka[1], Hiroshi Wada[2], Hidetoshi Eguchi[2], Takeshi Takahashi[3], Hiroshi Suemizu[3], Ryotaro Sakamori[1], Naoki Hiramatsu[1], Tomohide Tatsumi[1] and Tetsuo Takehara[1*].

[1] Department of Gastroenterology and Hepatology, Osaka University Graduate School of Medicine, Suita, Osaka, Japan

[2] Department of Gastroenterological Surgery, Osaka University Graduate School of Medicine, Suita, Osaka, Japan

[3] Department of Laboratory Animal Research, Central Institute for Experimental Animals, Kawasaki, Japan

Corresponding author: Tetsuo Takehara, M.D., Ph.D.

TEL: +81-6-6879-3621; FAX: +81-6-6879-3629

Email: takehara@gh.med.osaka-u.ac.jp

2-2 Yamadaoka Suita Osaka Japan 565-0871

Supplementary Figure 1.

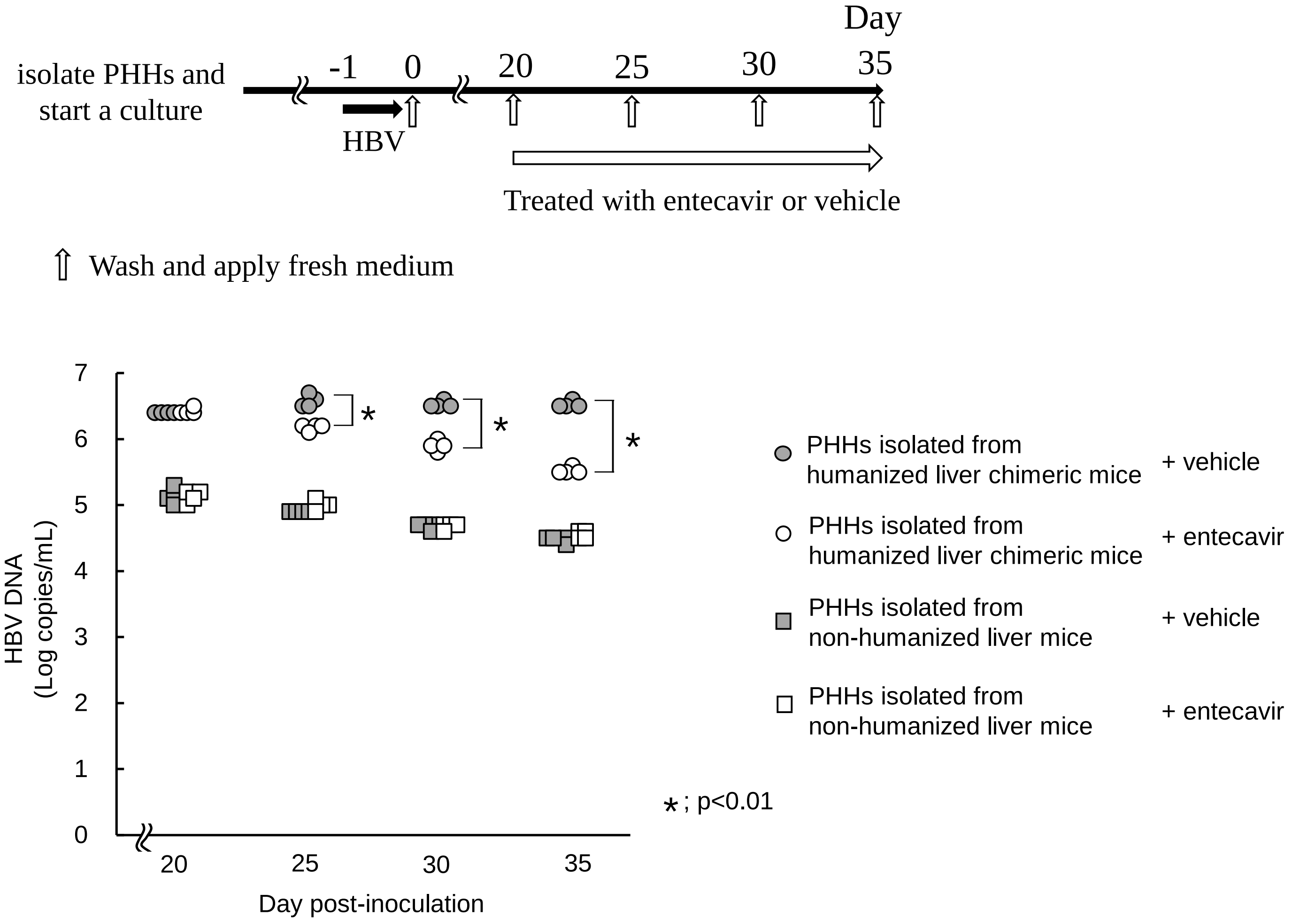

Supplementary Fig. 1

PHHs isolated from humanized liver chimeric mice or primary hepatocytes isolated from non-humanized liver mice were cultured with entecavir (1000 ng/mL) starting 20 days after HBV inoculation. HBV DNA levels in the culture medium (n=4).

Supplementary Figure 2.

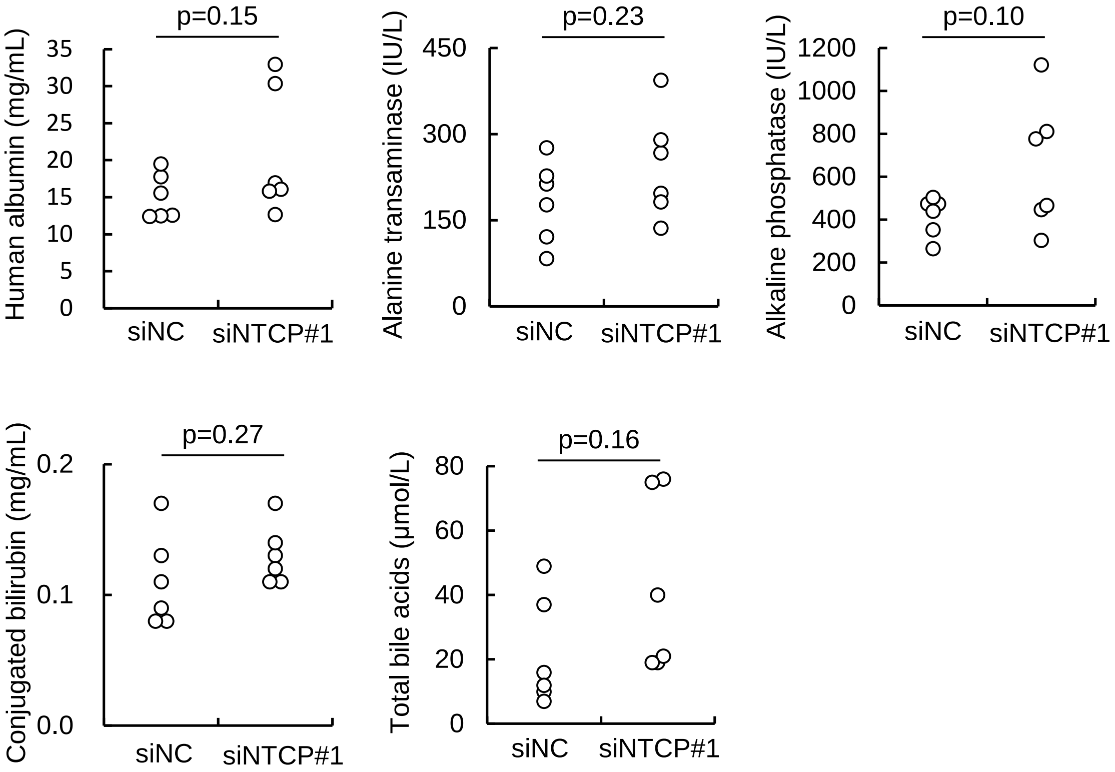

Supplementary Fig. 2

Humanized liver chimeric mice were transfected with siRNA against human-specific NTCP before and after HBV inoculation and sacrificed 2 weeks after inoculation (n = 6). Serum levels of human albumin, alanine transaminase, alkaline phosphatase, conjugated bilirubin and total bile acids.
